# Successful management of complete heart block in checkpoint inhibitor induced myocarditis by left bundle branch area pacing: a case report

**DOI:** 10.1093/ehjcr/ytae579

**Published:** 2024-11-15

**Authors:** Michael Meyers, Dragos Balf, Mohammad Q Raza, Xiaoke Liu

**Affiliations:** Mayo Clinic Health System, 800 West Ave. S, La Crosse, WI 54601, USA; Mayo Clinic, 200 1st St SW, Rochester, MN 55905, USA; Mayo Clinic Health System, 800 West Ave. S, La Crosse, WI 54601, USA; Mayo Clinic, 200 1st St SW, Rochester, MN 55905, USA; Mayo Clinic Health System, 800 West Ave. S, La Crosse, WI 54601, USA; Mayo Clinic, 200 1st St SW, Rochester, MN 55905, USA; Mayo Clinic Health System, 800 West Ave. S, La Crosse, WI 54601, USA; Mayo Clinic, 200 1st St SW, Rochester, MN 55905, USA

**Keywords:** Checkpoint inhibitor, Left bundle, Cardiac resynchronization, Heart block, Case report, Myocarditis

## Abstract

**Background:**

Optimal management of checkpoint inhibitor-induced complete heart block is unknown. Previous reports showed relatively high incidence of pacing failure due to the co-existing myocarditis.

**Case summary:**

A 71-year-old male with a prior history of stage IV metastatic squamous cell lung cancer presents was admitted for dyspnoea and hypotension 10 days after checkpoint inhibitor treatment using pembrolizumab. He was found to have myocarditis, third-degree AV block, severe left ventricular systolic dysfunction with EF 35%, and required pressure support. A dual chamber pacemaker using left bundle branch area pacing (LBBAP) was urgently placed that immediately improved his haemodynamics. Both the cathode and anode were able to capture the ventricle at different pacing outputs. The patient was taken off all intravenous pressors and successfully transferred to a larger centre for further management of the myocarditis with no further arrhythmia or hypotension.

**Discussion:**

In conclusion, because of the unique ability to capture a large amount of myocardium from both the tip and ring electrodes as well as the ability to deliver cardiac resynchronization therapy, LBBAP may be the preferred pacing strategy in patients who develop complete heart block due to checkpoint inhibitor-induced myocarditis.

**Learning points**

- Left bundle branch area pacing may be the preferred pacing strategy in complete heart block associated with check point inhibitor-induced myocarditis.
- Anodal capture may be a valuable back up pacing option in case of cathodal failure.

## Introduction

Immune checkpoint inhibitor myocarditis is a life-threatening condition characterized by lymphocytic myocardial infiltration and is classically associated with concomitant development of myasthenia gravis as well as myositis. Conduction system abnormalities were common with complete heart block (CHB) occurring in 15% of cases which could be fatal.^1–4^ The nature and optimal management of this often-malignant condition is poorly understood. Standard pacing therapy previously has been shown to be associated with potential lead malfunction including loss of capture, possibly related to the myocardial infiltration and/or inflammation leading to the inability to reliably capture the local myocardium surrounding the tip of the traditional pacemaker lead.^4^ In this case, we present evidence that left bundle area pacing may be the optimal pacing approach in heart block associated with myocarditis induced by checkpoint inhibitors.^5,6^

## Summary figure

**Figure:**
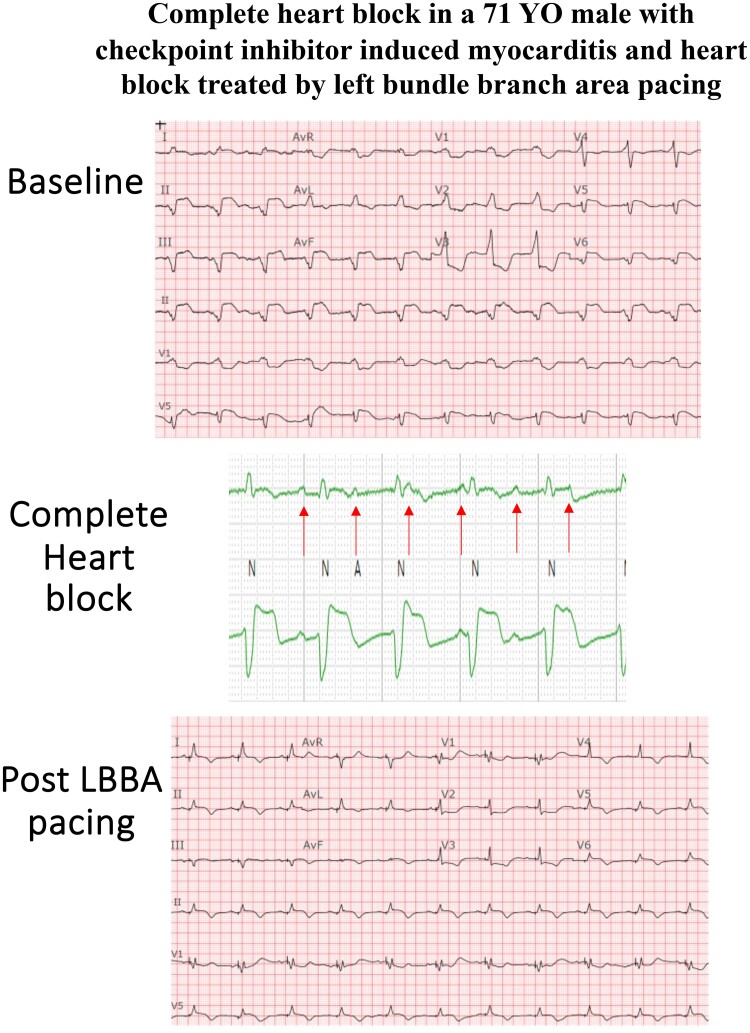


## Case report

**Figure 1:**
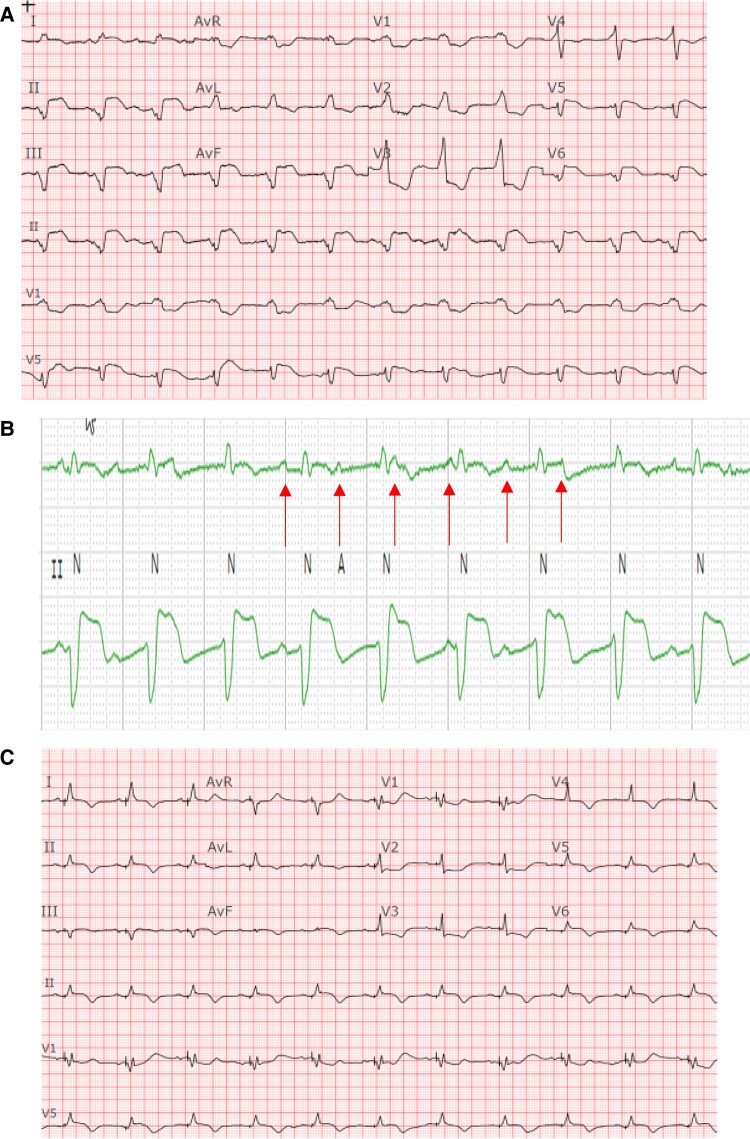
Baseline ECG demonstrating complete heart block with underlying accelerated junctional rhythm in a bifascicular block pattern in comparison to ECG post-pacing. (*A*) Baseline 12-lead ECG, suggestive of infra-hisian block given the very wide QRS in a bifascicular pattern indicative of diffuse conduction system abnormality. (*B*) Rhythm strips zoomed in to reveal the AV dissociation. (*C*) ECG post-pacing. An incomplete RBBB pattern with a narrower QRS complex.

A 71-year-old male with stage IV squamous cell lung cancer and diffuse bony metastases on pembrolizumab (last dose about 10 days prior to admission) was admitted for dyspnoea and hypotension. His past medical history was significant for chronic obstructive pulmonary disease, obesity, prior episode of diplopia with positive myasthenia antibodies, and recent ST-elevation myocardial infarction (MI) diagnosed at an outside institution. Physical examination was notable for signs of heart failure including elevated jugular venous pressure, bibasal/lower-zone crepitations over the lung fields, and bilateral lower extremity oedema. His laboratory testing showed elevated troponin T level at 2022ng/L (upper limit of reference range 15 ng/L) and NT-proBNP level of 3608 pg/mL (upper limit of reference range: 540 pg/mL). Cardiac echocardiogram demonstrated severe left ventricular systolic dysfunction with ejection fraction 35%. The patient was initially diagnosed and treated for acute MI from underlying coronary artery disease and had coronary stenting to distal circumflex artery. His symptoms continued to deteriorate and was admitted to our hospital, where he was diagnosed with checkpoint inhibitor-induced myositis, myocarditis, and myasthenia gravis. His hospital course was further complicated by the development of a third-degree heart block (*Figure 1*). Despite supportive medical therapy including high dose steroids and intravenous immunoglobulin, he continues to have CHB and required i.v. fluids as well as epinephrine infusion at a rate of 5–10 μg/min (for ∼24 h) to maintain systemic perfusion. Because of the persistent heart block and haemodynamic instability, he was urgently brought to the EP lab for pacemaker implantation and cardiac resynchronization therapy (CRT) through either conventional bi-ventricular pacing or conduction system pacing via left bundle branch area (LBBA) pacing. Because of the unclear prognosis (cumulative survival predicted to be <32% in one year^7^) and patient’s preference to not pursue cardiac resuscitation, only pacemaker (CRT-P) instead of defibrillator (CRT-D) was considered.

**Figure 2:**
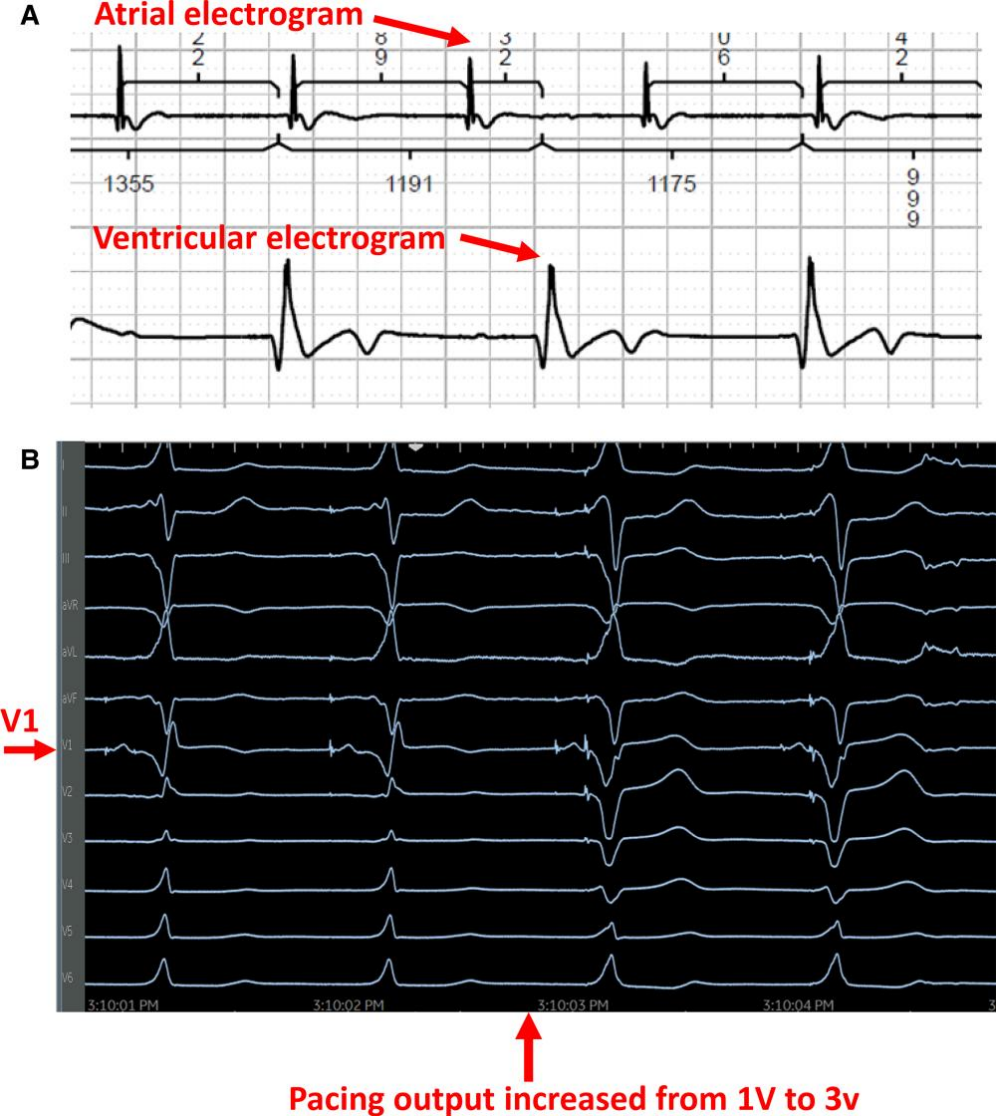
Intraoperative recordings during left bundle branch area pacing. (*A*) Intracardiac recording from the atrium as well as the ventricle demonstrating underlying complete heart block. (*B*) As pacing output increased from 1 to 3 V (tip to ring bipolar pacing configuration), the QRS transitioned from a narrow RBBB (cathodal tip pacing near LV endocardium) to a wide LBBB pattern consistent with capture from the anodal ring electrode near the RV endocardium.

**Figure 3:**
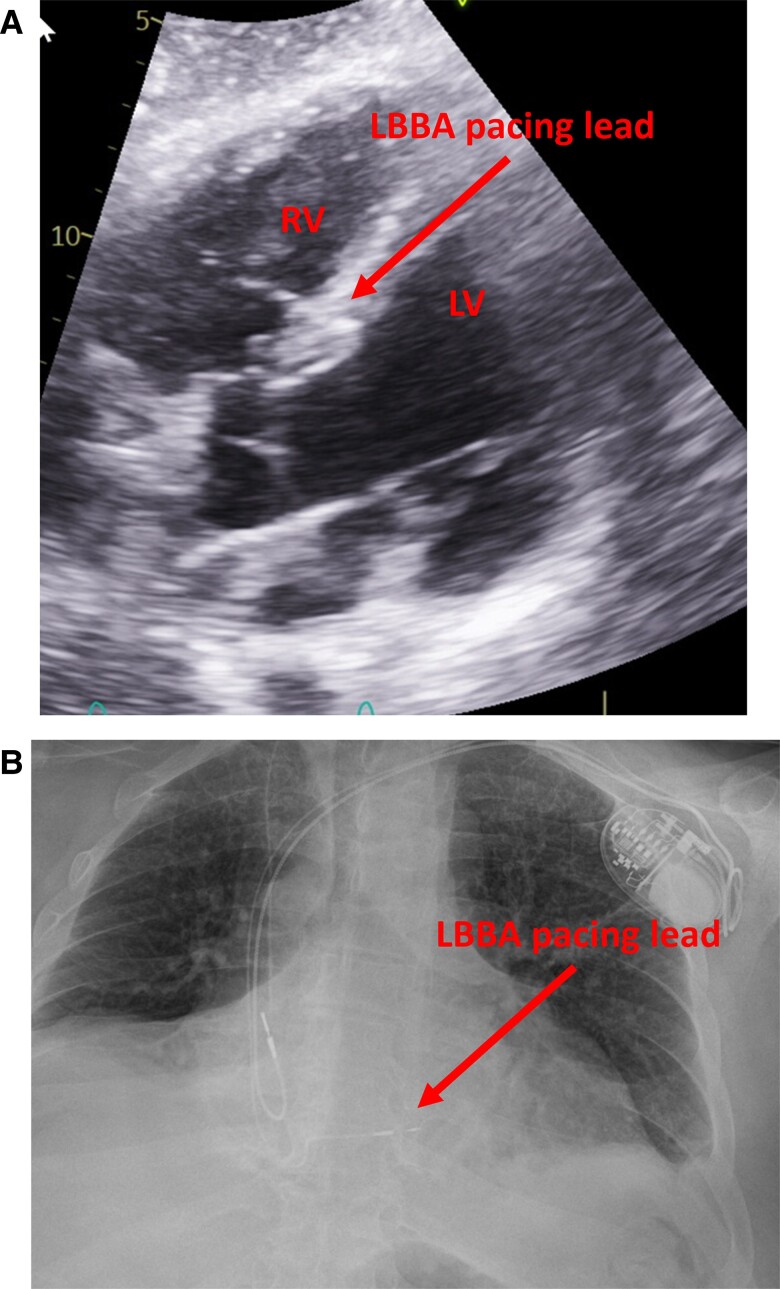
Post-op imaging demonstrating expected lead position. (*A*) ECHO imaging showing the ventricular lead deeply embedded in the basal ventricular septum with the tip abutting the LV endocardium. (*B*) Post-operative X-ray showing the septal location of the RV lead.

The patient was taken to the electrophysiology laboratory for pacemaker implantation to achieve CRT-P as well as to establish AV synchrony. The LBBAP technique was used because his pacing modality has been shown to offer excellent lead stability, achieves CRT with only two leads, and has the ability to capture the myocardium from both the cathode as well as the anode to ensure local myocardial capture.^8–10^ Through the axillary vein access, a Medtronic C315 delivery sheath (Medtronic, Inc., Minneapolis, MN) was advanced to the RV septum in the vicinity of the LBBA (1.5–2 cm distal to the tricuspid annulus towards the ventricular apex). A Medtronic 3830 lead (Medtronic, Inc., Minneapolis, MN) was advanced over the delivery sheath and penetrated the ventricular septum to engage the LBB. At the final location, pacing demonstrated abrupt conversion of the paced QRS from a left bundle branch block (LBBB) morphology to a much narrower right bundle branch block (RBBB) morphology. An LV activation time (measured from pacing stimulus to R wave peak or stimulation-R wave peat time) in V6 was 80 ms and a paced QRS duration of 104 ms suggested recruitment of the left bundle-Purkinje system. Pacing in the bipolar configuration from the tip to ring electrodes showed evidence of both cathodal (at lower pacing output) as well as anodal capture at a pacing output > 3 V at 0.5 ms.^11^ This was demonstrated by an abrupt change in the paced QRS morphology from a narrow RBBB morphology to a wide LBBB morphology when the pacing output was increased from 1 to 3 V, as shown in *Figure 2*. This is indicative of the ability to capture both sides of the septal myocardium including the LV endocardium that is near the tip electrode and the RV endocardium that is near the ring electrode. The procedure was completed successfully without complications. The procedure time from lidocaine injection to completion of pocket closure was 65 min with a fluoroscopy time of 5.9 min. A post-operative chest X-ray showed satisfactory lead position and an echocardiogram showing LBBA pacing lead seated appropriately in the deep septum (*Figure 3*). The pacemaker was programmed with initial output set at 2.5 V in the bipolar pacing configuration to allow CRT through LBBA pacing. The auto capture feature was turned on to allow backup anodal capture at a higher pacing output in case of cathodal capture failure.

The patient’s haemodynamics immediately improved after the dual chamber permanent pacemaker implant with conduction system pacing through LBBA pacing. He was taken off intravenous pressors and continued to do well from the cardiac standpoint. No rhythm or device related issues throughout the remainder of the hospital stay. He was subsequently transferred to a larger referral centre for further management of his respiratory status and ongoing myocarditis.

Remote monitoring in the first 3 weeks after the pacemaker implant did not show any evidence of device abnormalities. The patient eventually died one month after pacemaker implantation due to respiratory failure.

## Discussion

Complete heart block secondary to checkpoint inhibitor therapy is common and could be life-threatening. It should be considered in patients presenting with symptoms of myositis, myocarditis, elevated troponin, and prolonged QRS duration.^1–4^ Previous studies suggest that conventional RV pacing may be associated with an increased risk of lead malfunction including loss of capture, potentially due to the well-known myocarditis and myocardial inflammation associated with checkpoint inhibitor therapy.^5^ Traditional active fixation ventricular pacing leads are designed with a small helix that penetrates the septal myocardium to avoid perforation. However, if the local myocardium is affected by the inflammatory process from the myocarditis and becomes oedematous, it may lead to electrical abnormalities such as the inability to be captured by the pacemaker lead.

Left bundle branch area pacing has recently emerged as a promising alternative to RV apical pacing as well as CRT.^6,7,9,10^ To our knowledge, this is the first reported case demonstrating successful management of CHB in checkpoint inhibitor-induced myocarditis using LBBA pacing. The deep septal location, with the lead tip deeply embedded within the ventricular myocardium, offers potential advantages such as stable lead position, excellent pacing threshold and sensing.^9–11^ Moreover, anodal capture is frequently encountered with this pacing strategy.^12^ As the tip of the LBBA pacing lead penetrates deep into the septal myocardium, the anodal ring electrode frequently comes in close contact or partially penetrates the RV septal myocardium. This allows for capture of the RV endocardium as the distance between the helix and ring electrodes in the 3830 lead is only 9 mm^10^ while the thickness of the septal myocardium is typically 10–13 mm (14 mm in this case).^13^ Consequently, anodal capture of the septal myocardium is frequently observed during LBBA pacing.^12^ This feature may confer additional safety to ensure reliable capture of a larger bulk of the ventricle as capture can be achieved through both the tip and the ring electrodes, instead of the tip alone. Even if the myocardium around the tip electrodes becomes affected by the myocarditis process and loses the ability to be captured, the ring electrode which is in contact with a larger amount of myocardium due to the larger size may still be able to offer backup capture and avoid catastrophic ventricular asystole. This backup capture strategy can be easily achieved with the auto capture feature of modern pacemakers. Traditional active fixation pacemaker leads, on the other hand, only have a very small helix screwed into the apical myocardium and the proximal electrode is typically left in the bloodstream, not in close contact with the myocardium. Therefore, it is rare to have anodal capture with conventional active fixation, right ventricular pacing leads placed in the apical region. However, this can occur in cases of lead perforation, when the ring electrode is in close contact with the myocardium while the tip electrode completely penetrates and perforates the ventricular apex.^14^

The other advantage of the LBBA pacing is the ability to deliver CRT-P with only two leads as conventional CRT-P in this patient would require three leads including a CS lead, which may increase the complexity and risk of complication in this case. The ability to maintain AV synchrony and to achieve CRT simultaneously likely contributed to the immediate haemodynamic improvement post-procedure.

In conclusion, because of the unique ability to capture a large amount of myocardium from both the tip and ring electrodes, stable lead position with excellent pacing threshold as well as the ability to deliver CRT and avoid RV pacing induced cardiomyopathy, LBBA pacing may be an excellent pacing strategy in patients who develop CHB due to checkpoint inhibitor-induced myocarditis. Larger studies are required to further confirm this finding.

## Data Availability

Data are available upon reasonable request.

## Lead author biography

Dr Xiaoke Liu is a clinical cardiac electrophysiologist practicing at the Mayo Clinic Health System at La Crosse, WI with joint appointment at Mayo Clinic, Rochester, MN.

**Consent:** Patient consent was obtained using the *European Heart Journal – Case Reports* consent form in compliance with COPE guidelines and was documented in our hospital’s electronic medical records.

**Funding:** None declared.
